# Effect of red ginseng beverage on menopausal symptoms in Chinese women: A randomized, double-blind, placebo-controlled clinical trial

**DOI:** 10.1016/j.jgr.2025.12.004

**Published:** 2025-12-13

**Authors:** XueYing Wang, Yi Yang, TianYu Zheng, ChengZhi Wang, SiYu Qu, HongMei Luo, Chi Xu, YanLing Li

**Affiliations:** a First Teaching Hospital of Tianjin University of Traditional Chinese Medicine, Tianjin, China; b National Clinical Research Center for Chinese Medicine Acupuncture and Moxibustion, Tianjin, China; c Scientific Research Center, Conbio Technology Group Co, Ltd, Tianjin, China; d Eco-city Hospital of Tianjin Fifth Central Hospital, Tianjin, China

**Keywords:** Red ginseng, Menopausal symptoms, Vasomotor indicators, Lipid profiles, Anti-fatigue

## Abstract

**Background:**

Menopause involves both physical and psychological changes, notably vasomotor and mood symptoms. Menopausal symptoms adversely affect women's quality of life and work. This study evaluates the effects of red ginseng beverage (RGB) on menopausal symptoms in Chinese women.

**Methods:**

This 90-day, randomized, double-blind, placebo-controlled trial involved 112 eligible women assigned to either an RGB or placebo group. Primary outcomes were the Kupperman Index. Secondary outcomes included vasomotor markers, psychological assessments, lipid profiles, exercise-induced fatigue test indicators and safety indicators. Safety assessments included fire-heat symptom scale, female hormone levels, endometrial thickness, breast ultrasound, and standard laboratory tests. All outcomes were assessed at baseline and upon study completion.

**Results:**

RGB group primary outcomes compared to placebo: (1) the *Kupperman Index* (KI) scores were significantly improved after test (*p* < 0.001), (2) the levels of serum nitric oxide (NO) increased significantly (*p* < 0.01), the content of and endothelin-1 (ET-1) decreased significantly (*p* < 0.01); the results of the secondary outcomes in RGB group: (1) *Beck Depression Inventory* (BDI) and *Athens Insomnia Scale* (AIS) scores decreased significantly (*p* < 0.001), (2) Total cholesterol and low-density lipoprotein cholesterol decreased significantly (*p* < 0.05), (3) Creatine kinase and blood lactate decreased significantly (*p* < 0.05), subjective physical strength rating scale scores decreased significantly (*p* < 0.01). No intergroup differences were observed in hormonal levels, endometrial thickness, urinalysis, hematology, or biochemistry. RGB administration showed no significant effect on *fire-heat symptom scores*.

**Conclusion:**

These findings demonstrate RGB's efficacy and safety for menopausal symptom management.

## Graphical abstract

**Figure:**
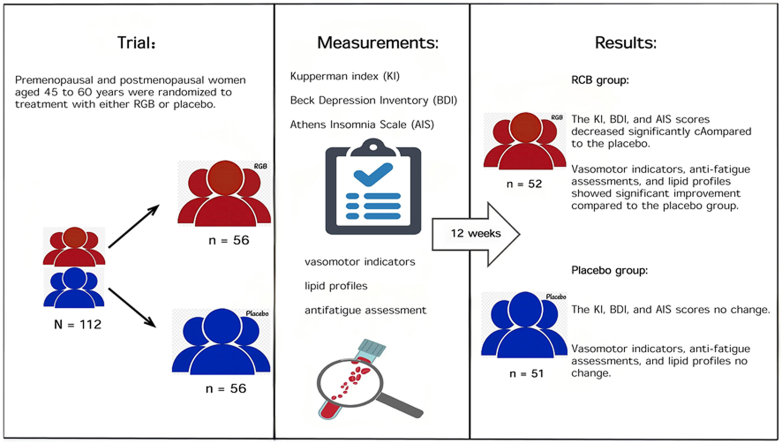

## 1 Introduction

Menopause refers to the period before and after the cessation of menstruation in women. During this period, women commonly present with vasomotor symptoms such as hot flashes and sweating; autonomic nervous system symptoms including sleep disturbances, palpitations, and fatigue; psychological symptoms like anxiety and depression due to decreased estrogen. Menopause is further associated with increased risk of osteoporosis, general joint and muscle pains, and cardiovascular disease [[1], [2], [3]]. It is estimated that by 2030 there will be approximately 210 million menopausal women in China, accounting for nearly 1/7 of the global menopausal population. Among them, over 85 % may experience varying degrees of menopausal symptoms, ranging from mild to severe manifestations [4]. Hormone replacement therapy containing estrogen is the effective primary treatment for menopausal symptoms [5]. However, long-term use of estrogen may increase the risk of venous thrombosis, breast cancer, and cardiovascular disease [6]. Complementary and alternative medicine has gained popularity as a natural approach to relieving menopausal symptoms [[7], [8], [9]].

Red ginseng, produced by steaming and drying fresh ginseng, contains ginsenosides and polysaccharides as its main bioactive constituents [10,11]. Red ginseng has been reported to possess diverse pharmacological effects, including enhancing immunity, relieving fatigue, improving memory, enhancing blood circulation, antioxidation, ameliorating insulin resistance and improving menopausal symptoms in women [[12], [13], [14], [15]]. Numerous clinical studies have confirmed that red ginseng can improve menopausal symptoms in postmenopausal women. Multiple high-quality randomized controlled trials (RCTs) have shown that daily supplementation of red ginseng for 12 weeks can significantly reduce KI scores and alleviate hot flashes [16]. Studies suggest that red ginseng has an improving effect on sexual function in premenopausal and postmenopausal women [[17], [18], [19]]. In addition, red ginseng can help alleviate depression and fatigue symptoms in postmenopausal women, and has a positive impact on lipid profiles [16,17,20]. Notably, Red ginseng intake has not been shown to affect estrogen levels or endometrial thickness in postmenopausal women [21,22]. However, there are still some limitations in this research field. The study participants are mainly focused on postmenopausal women, with relatively insufficient attention paid to the perimenopausal stage. Additionally, there is a lack of in-depth studies specifically targeting the Chinese population, and generalizability of the conclusions needs further verification. Finally, the mechanism by which red ginseng improves menopausal symptoms has not been fully elucidated. Therefore, we conducted this study to investigate the effects of red ginseng beverage in Chinese perimenopausal and postmenopausal women, and to explore the underlying mechanisms of its action.

## 2 Materials and methods

The evaluation methods including inclusion/exclusion criteria and evaluation indicators align with the Amendment of Health Functional Food Code issued by Ministry of Food and Drug Safety (MFDS, Korea) [23].

### 2.1 Ethical approval and trial registration

This study was approved by the Medical Ethics Committee of First Teaching Hospital of Tianjin University of Traditional Chinese Medicine (Approval No. TYLL 2023K-046). The study was conducted in accordance with the Declaration of Helsinki, and informed consent was obtained from all participants. The trial was prospectively registered at the Chinese Clinical Trial Registry on January 6, 2024 (registration number: ChiCTR2400079570; URL: https://www.chictr.org.cn/index.html). The trial was conducted at the outpatient clinic of the First Teaching Hospital of Tianjin University of Traditional Chinese Medicine.

### 2.2 Materials

RGB and placebo were offered by the Korea Ginseng Corporation. RGB appears as dark brown liquid, supplied in 10g per pack. Each pack contained water, concentrated red ginseng extract (2.1 g), sodium citrate, and stevioside. The placebo was identical to the RGB in appearance, taste, packaging, and dosage form but lack active red ginseng extract. Subjects were unable to distinguish between the placebo and RGB. The placebo (10 g) contained water, maltose, denatonium benzoate, red ginseng flavor, and sodium citrate.

### 2.3 Study population

#### 2.3.1 Inclusion criteria

(1) premenopausal and postmenopausal women aged 45–60 years; (2) follicle-stimulating hormone (FSH) level ≥30 mIU/mL; (3) Kupperman Index (KI) score >25 with at least two of the following symptoms: insomnia, irritability, depression, or fatigue; (4) signing of informed consent.

#### 2.3.2 Exclusion criteria

(1) body mass index >30 kg/m^2^; (2) women who have used female hormones or similar hormone preparations within the past three months; (3) women diagnosed with endometrial hyperplasia, uterine cancer, endometrial cancer, breast cancer or breast diseases, or sex hormone related diseases; (4) women with a history of cancer; (5) patients undergoing hysterectomy; (6) women meeting any of the following criteria within the past year: severe migraine, thromboembolism, cerebrovascular disease, myocardial infarction, angina pectoris or history of coronary artery surgery; (7) women who have received drugs for the treatment of hyperlipidemia or cardiovascular disease within the past three months; (8) women with serious mental disorders such as depression and anxiety, or those undergoing current treatment with antidepressants, sleeping pills, or other psychotropic medications; (9) patients receiving oral or injectable therapeutic agents for abnormal uterine bleeding and related diseases after menopause; (10) hypertensive patients with blood pressure >160/100 mmHg; (11) patients with uncontrolled diabetes (fasting blood glucose >180 mg/dL or restarting diabetes medication within the past three months); (12) thyroid disease patients with thyroid stimulating hormone(TSH) levels <0.1 μU/mL or > 10 μU/mL; (13) individuals who abuse drugs or alcohol; (14) aspartate aminotransferase (AST) or alanine aminotransferase (ALT) exceeding three times the upper limit of normal values for research institution; (15) serum creatinine exceeding twice the upper limit of normal values for research institution; (16) individuals who have received thyroid hormone preparations, clonidine, anticoagulants, or antithrombotic agents within the past three months; (17) individuals who have taken medications or health functional foods targeting menopausal symptoms(including vitamins/antioxidants products, fatigue-relief formulations, cardiovascular health supplements) within the past 30 days; (18) individuals who have consumed red ginseng-related products within the past 3 months; (19) patients currently receiving bisphosphonate agents or other anti-osteoporosis medications; (20) vegetarians; (21) endometrial thickness >7 mm; (22) product adherence <80 %; (23) individuals deemed unsuitable for clinical trials by the principal investigator.

### 2.4 Study design

This study was conducted as a randomized double-blind placebo-controlled trial. The recruitment for this study was conducted from January to April 2024. A total of 112 subjects were randomly allocated to RGB or placebo group in a 1:1 ratio using random numbers. The random numbers were generated by an independent statistician. Allocation was concealed via sealed envelopes. The RGB group consumed one pack once a day for 90 days. The placebo group received placebo under identical conditions. Safety assessments, primary outcomes and secondary outcomes were evaluated at baseline and study completion. Participant adherence was assessed using self-reported diary cards and by quantifying residual study product in returned vials during follow-up visits.

### 2.5 Outcome measures

#### 2.5.1 Primary outcome

The primary outcome of this trial is the Kupperman Index (KI) [24].

#### 2.5.2 Secondary outcomes

Secondary outcomes included: (1) vasomotor indicators [NO, endothelial nitric Oxide Synthase(eNOS), and ET-1)]. (2) psychological assessments (BDI [25] and AIS [26]); (3) serum lipid profiles [total cholesterol (TC), triglycerides (TG), high-density lipoprotein cholesterol (HDL-C), and low-density lipoprotein cholesterol (LDL-C)]; (4) antifatigue assessment, comprising objective measures from a gradient load exercise (pre-exercise cortisol, post-exercise creatine kinase, blood lactate) and subjective physical strength rating scale; the detailed exercise protocol is available in Appendix A. (5) Safety assessments comprised: (a) heart rate and blood pressure; (b) hematological, urinalysis, and fecal testing; (c) kidney and liver function; (d) TSH, estradiol (E2), follicle-stimulating hormone (FSH), and luteinizing hormone (LH); (e) endometrial thickness and breast ultrasound examination; (f) electrocardiogram, chest radiography, and abdominal B-ultrasound examination (checked once before the test); (g) *fire-heat symptom scale* [27].

### 2.6 Statistical processing

#### 2.6.1 Sample size determination

The sample size was determined according to the Technical Specifications for Testing and Evaluation of Health Food issued by the China National Health Commission, which recommends a minimum of 50 participants per group for human intervention trials [28]. Subjects with product consumption exceeding 80 % were included in the final outcome analysis.

#### 2.6.2 Data statistical analysis

Statistical analyses were performed using SPSS (IBM Corp., Armonk, NY, USA) version 21.0. Paired t-tests were used to analyze self-controlled data. Independent t-tests were used to compare means of two groups after verifying variance homogeneity via Levene's test. When datasets violated normality or homogeneity of variance, appropriate data transformations were applied, and t-tests were performed on transformed data after satisfying normality and homogeneity of variance. If the transformed data still could not meet the requirement of normal variance homogeneity, *t*-test or rank sum test were used. The rank sum test was applied to data with square variances but large coefficients of variation (such as CV > 50 %). Effective rates were analyzed using Chi-square test.

## 3 Results

### 3.1 Characteristics of subjects

**Fig. 1:**
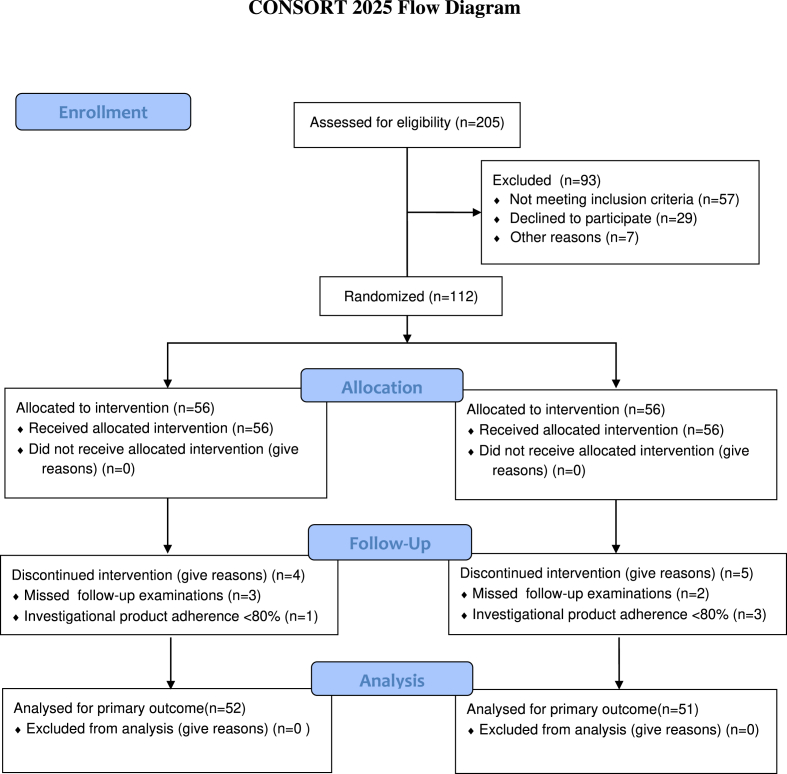
Consort diagram for the clinical Trial.

112 eligible subjects were screened using protocol-defined criteria. Nine women were excluded from the trial due to missed protocol-specified follow-up examinations or investigational product adherence <80 %. In the final outcome analysis, 52 subjects were included in the RGB group and 51 in the placebo group. The dropout rates for subjects in the RGB group and placebo group were 7.1 % and 8.9 % (see Fig. 1).

As shown in Table 1, baseline demographic characteristics, NO, eNOS, ET-1, and KI scores showed no significant differences between the two groups (*p* > 0.05), suggesting comparable baseline characteristics between the two groups.

**Table 1:** Baseline characteristics of the two groups before the test ($\bar{x}$ ±SD).

	RGB group(n = 52)	Placebo group(n = 51)
Age (years old)	53.37 ± 4.75	52.10 ± 4.29
BMI(kg/m^2^)	25.02 ± 2.98	24.76 ± 3.18
Systolic pressure(mm/Hg)	126.48 ± 11.28	123.08 ± 13.40
Diastolic pressure(mm/Hg)	77.62 ± 8.21	76.02 ± 9.25
Kupperman index	28.85 ± 2.58	28.63 ± 2.68
NO(μmoL/mL)	0.07 ± 0.02	0.06 ± 0.02
eNOS (ng/mL)	3.52 ± 1.50	3.25 ± 1.34
ET-1(pg/mL)	20.19 ± 5.28	21.64 ± 4.76
FSH(mIU/mL)	71.54 ± 25.60	65.16 ± 22.18

### 3.2 Primary outcome

As shown in Table 2, Kupperman index scores significantly decreased in the RGB group (*p* < 0.001) after 90 days. Compared with the placebo group, the Kupperman index scores in the RGB group significantly decreased, with significant improvements in key symptom items: hot flashes and sweating (*p* < 0.001), paresthesia (*p* < 0.05), insomnia (*p* < 0.001), irritability (*p* < 0.001), depression (*p* < 0.05), and fatigue (*p* < 0.001).

**Table 2:** Changes of total score of Kupperman index before and after the test (‾x±SD).

	RGB group(n = 52)		Placebo group(n = 51)	
	Before test	After test	Before test	After test
Total Score of Kupperman index	28.85 ± 2.58	19.88 ± 4.99∗∗∗###	28.63 ± 2.68	28.14 ± 4.68
Hot flashes and sweating	8.38 ± 2.28	5.15 ± 2.78∗∗∗###	8.31 ± 2.51	8.63 ± 2.81
Paresthesia	2.81 ± 1.33	1.85 ± 1.24∗∗∗#	2.47 ± 1.42	2.39 ± 1.39
Insomnia	3.04 ± 1.08	1.50 ± 1.18∗∗∗###	2.86 ± 1.08	2.75 ± 1.13
Irritability	3.42 ± 1.21	1.88 ± 1.28∗∗∗###	3.33 ± 1.11	3.14 ± 1.15
Depressed	0.85 ± 0.64	0.67 ± 0.51∗∗#	0.92 ± 0.52	0.94 ± 0.54
Vertigo	1.15 ± 0.54	1.12 ± 0.51	1.06 ± 0.58	1.02 ± 0.58
Fatigue	1.65 ± 0.56	0.88 ± 0.65∗∗∗###	1.61 ± 0.49	1.59 ± 0.61
Bone joint, and muscle pain	1.04 ± 0.59	1.00 ± 0.40	1.22 ± 0.58	1.20 ± 0.66
Headache	1.23 ± 0.58	1.19 ± 0.56	1.12 ± 0.62	1.10 ± 0.61
Cardiopalmus	1.04 ± 0.39	0.92 ± 0.48	1.06 ± 0.42	1.02 ± 0.55
Formication	0.81 ± 0.63	0.63 ± 0.63∗∗	0.82 ± 0.56	0.76 ± 0.59
Algopareunia	1.73 ± 1.12	1.62 ± 1.05	2.04 ± 1.47	1.92 ± 1.50
Urinary symptoms	1.69 ± 1.39	1.46 ± 1.20	1.80 ± 1.28	1.69 ± 1.29

Comparison within group ∗∗*p* < 0.01 ∗∗∗*p* < 0.001,comparison between groups #*p* < 0.05 ###*p* < 0.001.

### 3.3 Secondary outcomes

#### 3.3.1 Vasomotor indicators

As shown in Table 3, after 90-day intervention, the RGB group showed a significant increase in serum NO, alongside a significant decrease in ET-1, while eNOS activity showed no significant alteration. Compared with the placebo group, the RGB group had significantly increased NO levels (*p* < 0.01) and decreased ET-1 concentrations (*p* < 0.05), with no intergroup difference in eNOS activity.

**Table 3:** Changes of secondary outcomes before and after the test (‾x±SD).

	RGB group(n = 52)		Placebo group(n = 51)	
	Before test	After test	Before tes	After test
**Vasomotor indicators**				
NO(μmoL/mL)	0.07 ± 0.02	0.08 ± 0.02∗##	0.06 ± 0.02	0.06 ± 0.02
eNOS (ng/mL)	3.52 ± 1.50	3.33 ± 1.01	3.25 ± 1.34	3.21 ± 1.06
ET-1(pg/mL)	20.19 ± 5.28	18.10 ± 5.12∗##	21.64 ± 4.76	21.38 ± 5.20
**Psychological assessments**				
Beck depression inventory	13.87 ± 2.53	10.83 ± 2.46∗∗∗###	13.55 ± 3.07	13.76 ± 3.56
Athens insomnia scale	5.52 ± 1.74	4.40 ± 1.38∗∗∗###	5.92 ± 1.51	6.10 ± 1.40
**Lipid profiles**				
TC	6.27 ± 1.17	5.34 ± 0.93∗∗∗#	5.97 ± 1.14	5.78 ± 1.20
TG	1.27 ± 0.67	1.37 ± 0.70	1.30 ± 0.74	1.38 ± 0.79
HDL-C	1.71 ± 0.33	1.64 ± 0.37	1.73 ± 0.31	1.65 ± 0.36
LDL-C	3.57 ± 0.84	3.06 ± 0.76∗∗∗#	3.28 ± 0.85	3.41 ± 0.86

Comparison within groups ∗ *p* < 0.05,comparison between groups ##*p* < 0.01.

#### 3.3.2 Beck Depression Inventory, and Athens Insomnia Scale

As shown in Table 3,the RGB group demonstrated significant reductions in BDI and AIS scores compared to both its own baseline and the placebo group (*p* < 0.001).

#### 3.3.3 Lipid profiles

As shown in Table 3, compared with baseline, serum TC and LDL-C significantly decreased in the RGB group (*p* < 0.001). Compared with the placebo group, the RGB group exhibited decreases in TC and LDL-C levels (*p* < 0.05). TG and HDL-C remained stable throughout the study, with no statistically significant alterations detected in intra-group or inter-group comparisons.

#### 3.3.4 Antifatigue assessment

The results of the gradient load exercise test showed that compared with the placebo group, the RGB group exhibited better physiological recovery exercise, characterized by significant reductions in blood lactate and creatine kinase levels as well as a decrease in subjective physical strength rating scores (All detailed data are available in Table S1 of Appendix A).

#### 3.3.5 Results of safety assessments

As shown in Table 4, the results of hematological, urinalysis, fecal, liver, and kidney function tests for both groups remained largely within the normal range before and after the trial, with no significant changes observed (*p* > 0.05). It is noteworthy that the fasting blood glucose (FBG) levels in the RGB group showed a significant decrease compared to baseline. No significant changes were observed in *fire-heat symptom scores* following RGB administration. When comparing the RGB group at the study completion to its own baseline and to the placebo group, there were no significant differences in female hormones, TSH, or endometrial thickness (*p* > 0.05). No allergic reactions or adverse events were reported during the trial period.

**Table 4:** Changes of safety assessments before and after the test ($\bar{x}$ ±SD).

	RGB group(n = 52)		Placebo group(n = 51)	
	Before test	After test	Before test	After test
Red blood cell count( × 10^12^/L)	4.49 ± 0.36	4.44 ± 0.30	4.52 ± 0.30	4.48 ± 0.34
White blood cell count( × 10^9^/L)	5.52 ± 1.27	5.34 ± 1.45	5.38 ± 1.71	5.23 ± 1.27
Platelet count( × 10^9^/L)	258.44 ± 57.71	247.88 ± 57.66	260.10 ± 50.42	255.37 ± 49.25
Hemoglobin (g/L)	131.37 ± 13.34	130.81 ± 11.78	134.53 ± 10.69	132.51 ± 11.91
Total protein(g/L)	81.38 ± 4.46	79.36 ± 9.09	80.90 ± 4.74	79.20 ± 9.20
Albumin(g/L)	46.96 ± 1.77	46.59 ± 3.07	47.11 ± 2.32	46.92 ± 4.93
ALT(U/L)	21.56 ± 15.95	19.17 ± 8.89	22.59 ± 16.77	23.90 ± 23.21
AST(U/L)	24.06 ± 11.93	24.65 ± 11.81	25.39 ± 11.60	27.00 ± 14.25
Urea(mmol/L)	5.01 ± 1.15	4.75 ± 0.99	4.93 ± 1.14	4.59 ± 1.07
Creatinine(μmol/L)	61.35 ± 8.70	60.60 ± 8.70	60.39 ± 9.92	58.45 ± 7.75
Uric acid(μmol/L)	256.21 ± 70.81	258.92 ± 60.00	241.49 ± 62.09	242.78 ± 63.20
FBG(mmol/L)	5.69 ± 0.58	5.31 ± 0.56∗∗∗	5.80 ± 0.99	5.70 ± 1.63
Urinalysis	Normal	Normal	Normal	Normal
Fecal test	Normal	Normal	Normal	Normal
Systolic pressure(mm/Hg)	126.48 ± 11.28	126.31 ± 11.57	123.08 ± 13.40	122.98 ± 13.66
Diastolic pressure(mm/Hg)	77.62 ± 8.21	77.48 ± 7.73	76.02 ± 9.25	75.51 ± 9.26
Heart rate(times/minute))	69.75 ± 9.99	70.15 ± 8.06	71.20 ± 8.33	71.43 ± 7.30
E2 (pmol/L)	151.17 ± 298.19	164.06 ± 301.59	156.18 ± 308.09	196.61 ± 337.49
FSH (mIU/mL)	71.54 ± 25.60	67.94 ± 29.90	65.16 ± 22.18	63.79 ± 31.41
LH (mIU/mL)	26.55 ± 17.36	28.94 ± 18.39	28.80 ± 19.47	30.97 ± 20.29
TSH(mIU/L)	3.01 ± 1.63	3.21 ± 4.35	3.29 ± 1.95	2.96 ± 2.15
Endometrial thickness (cm)	0.38 ± 0.18	0.37 ± 0.26	0.39 ± 0.17	0.36 ± 0.25
Fire-heat symptoms scale	41.50 ± 11.87	42.08 ± 10.37	42.31 ± 11.58	42.78 ± 11.72

Comparison within groups∗∗∗*p* < 0.001.

## 4 Discussion

In the present study, we comprehensively assessed the efficacy and safety of red ginseng for menopausal symptoms in Chinese women. Our results show that there was no significant changes in female estrogen levels, and endometrial thickness after taking RGB for 90 days, which aligns with previous clinical studies [16,22]. However, another experiment demonstrated that red ginseng extract activates estrogen receptors in cells [29], suggesting that red ginseng may possess estrogenic activity but is insufficient to induce a physiological response in vivo.

There is concern that long-term use of red ginseng can induce "Shanghuo" (fireness). Our research results showed that there was no significant increase in the fire-heat symptom scale scores, and this clinical study [30] is consistent with our findings. Notably, while the cited study implemented a 4-week red ginseng intervention [30], our research has been extended to 90 days. No significant changes were observed in the other safety indicators including breast ultrasound, hematologic and blood chemistry tests after taking RGB for 90 days. No adverse events related to the RGB occurred during the test. These findings on the safety of red ginseng in our study are consistent with previous literature [16,22], but based on this, we innovatively added the "fire-heat symptom scale score" as a culture-related safety indicator.

Our results demonstrated that the KI scores in the RGB group were significantly lower than those in the placebo group, and the symptoms of hot flashes and sweating improved. A previous clinical study [16] also showed the same results, but the difference is that our research results also showed improvement in other symptoms including paresthesia, sleep disturbances, irritability, depression, and fatigue. The superior intervention effect observed in this study may be attributed to the following three key factors. First, our study included the perimenopausal population, who may be more sensitive to interventions. Second, there are differences in population background: all participants in this study were Chinese women, and existing literature indicates that common menopausal symptoms among Chinese women include fatigue, weakness, irritability, sleep disturbances, musculoskeletal joint pain, and hot flashes and sweating. In contrast, hot flashes and sweating - the most common symptom among western women - rank only 5th in Chinese women [31], indicating that our intervention may be more precisely aligned with the core symptom profile of the study population. Finally, our relatively larger sample size (n = 103 in this study vs. n = 69 in Reference 16) may have provided greater statistical power, enabling us to detect subtle symptomatic improvement effects.

NO is an endothelium-dependent relaxing factor widely distributed in tissues and cells throughout the body. NO is synthesized through NOS-catalyzed oxidation of L-arginine. After production, NO diffuses into target cells to exert biological effects, including regulating vascular tone through its effects on endothelial and smooth muscle cells, ultimately inducing vasodilation [32]. ET-1, a 21-amino acid vasoactive peptide secreted by vascular endothelial cells, is a potent vasoconstrictor that contributes to the pathogenesis of atherosclerosis, coronary artery disease, and hypertension [33]. This study demonstrated that the RGB group exhibited significantly higher NO levels and significantly lower ET-1 levels compared with the placebo group. These suggest that RGB may promote vasodilation. Notably, these vascular biomarker changes were associated with improvement of hot flashes and sweating. Compared with the similar randomized clinical trials (RCTs) previously [16,22], this study has further revealed the potential mechanism by which red ginseng improves menopausal symptoms—by regulating NO and ET-1 to improve vascular endothelial function. Existing studies provide indirect support for this mechanism. A randomized controlled trial showed that red ginseng extract can significantly improve flow-mediated vasodilation [34]. Another clinical trial showed that red ginseng extract increases NO concentrations in exhaled breath [35]. These studies confirm the regulatory effect of red ginseng on blood vessels.

Vasomotor symptoms, insomnia, and depression during menopause are interconnected and synergistically impact women's health [36]. Depressive symptoms were assessed using the 21-item BDI, while sleep quality was measured with the 8-item AIS to evaluate the efficacy of RGB intervention in menopausal women. Our research results found that RGB intervention can significantly reduce BDI and AIS scores, consistent with improvements of depressive and insomnia symptoms in KI. Wiklund IK et al. found that compared with the placebo group, the ginseng group showed a significant improvement in the depression subset of the Psychological General Well-Being (PGWB) index, while the anxiety showed no improvement [17]. The improvement in depressive status observed in our study is consistent with the earlier findings of Wiklund et al. While the results regarding anxiety were inconsistent, this may be due to the use of different assessment tools. Compared with the similar randomized clinical trials (RCTs) previously [16,22], we conducted an additional assessment of the impact of red ginseng on insomnia and depression in menopausal populations.

This study found that compared with the placebo group, blood lactate, creatine kinase levels and subjective physical strength rating scores in the RGB group were significantly reduced, indicating that RGB exhibited anti-fatigue efficacy in the standardized gradient load exercise test. This study using a standardized exercise test to evaluated the efficacy of RGB in alleviating physical fatigue in menopausal women. Menopausal fatigue is mainly attributed to a series of neurophysiological changes caused by a decrease in estrogen levels, including brain energy metabolism, mitochondrial function, HPA axis dysfunction, oxidative stress, and upregulation of inflammatory markers [[37], [38], [39]]. This is also the physiological basis involved in exercise-induced fatigue [40]. Previous studies have demonstrated that red ginseng can improve not only exercise-induced fatigue but also alleviate menopause fatigue in postmenopausal women [20,41].

Dyslipidemia can worsen menopausal symptoms, which is particularly evident in cases of high triglycerides (TG) [42,43]. It also elevates cardiovascular disease risk in menopausal women [44]. The pathogenesis is primarily attributed to estrogen deficiency and dysregulated lipid metabolism characteristic of the menopausal transition [45]. Our study demonstrated that RGB intervention alleviated menopausal symptoms while exerting selective effects on blood lipids. Specifically, RGB significantly reduced TC and LDL-C levels. However, it did not significantly alter TG and HDL-C levels. These findings suggest that RGB operates through multiple pathways, which may function independently or synergistically. First, the improvement in menopausal symptoms is likely mediated through direct regulation of vascular function. Our study confirmed that RGB restores the balance between NO and ET-1, a pathway not closely associated with TG metabolism. Second, RGB reduces TC and LDL-C by downregulating the expression of HMGCR and SREBP2, thereby inhibiting cholesterol synthesis in HepG2 cells [46]. Therefore, the alleviation of menopausal symptoms and the specific improvements in lipid profiles represent distinct outcomes, collectively illustrating the multi-targeted effects of RGB.

This study has several limitations. First, the safety of red ginseng products requires further validation through extended administration periods or larger sample sizes. Second, the inclusion of women with different Traditional Chinese Medicine (TCM) body constitution types may confound the conclusion that "taking RGB for 3 months did not result in Shanghuo (fireness symptoms)". There are differences in the reactions of women with different TCM body constitution types to red ginseng. The next step of the study is to apply metabolomics to elucidate the mechanism by which red ginseng alleviates menopausal symptoms.

## Declaration of competing interest

The authors whose names are listed immediately below certify that they have NO affiliations with or involvement in any organization or entity with any financial interest (such as honoraria; educational grants; participation in speakers’ bureaus; membership, employment, consultancies, stock ownership, or other equity interest; and expert testimony or patent-licensing arrangements), or nonfinancial interest (such as personal or professional relationships, affiliations, knowledge or beliefs) in the subject matter or materials discussed in this manuscript.

## References

[1] Chinese Medical Doctor Association (CMDA)'s General Practitioners Sub-association,The Primary Care Branch of Beijing Institute of Obstetrics and Gynecology (2021). Consensu-son health management in climacteric women in primary medical institutions edition. Chinese General Practice.

[2] Koothirezhi R., Ranganathan S. (2025). Postmenopausal syndrome. StatPearls Treasure Island (FL).

[3] Santoro N., Epperson C.N., Mathews S.B. (2015). Menopausal symptoms and their management. Endocrinol Metab Clin N Am.

[4] Hill K. (1996). The demography of menopause. Maturitas.

[5] Cobin R.H., Goodman N.F., Committee A.R.E.S. (2017). American association of clinical endo-crinologists and American college of endocrinology position statement on Menopause-2017 update. Endocr Pract.

[6] Levy B., Simon J.A. (2024). A contemporary view of menopausal hormone therapy. Obstet Gynecol.

[7] Smith-Francis M.J. (2024). Complementary and alternative medicine for menopause. Nurs Clin.

[8] Mehrnoush V., Darsareh F., Roozbeh N., Ziraeie A. (2021). Efficacy of the complementary and alternative therapies for the management of psychological symptoms of menopause: a systematic review of randomized controlled trials. J Menopausal Med.

[9] Posadzki P., Lee M.S., Moon T.W., Choi T.Y., Park T.Y., Ernst E. (2013). Prevalence of complementary and alternative medicine (CAM) use by menopausal women: a systematic review of surveys. Maturitas.

[10] Ye X.W., Li C.S., Zhang H.X., Li Q., Cheng S.Q., Wen J. (2023). Saponins of ginseng products: a review of their transformation in processing. Front Pharmacol.

[11] Han M.J., Kim D.H. (2020). Effects of red and fermented ginseng and ginsenosides on allergic disorders. Biomolecules.

[12] Hyun S.H., Kim S.W., Seo H.W., Youn S.H., Kyung J.S., Lee Y.Y. (2020). Physiological and pharmacological features of the non-saponin components in Korean red Ginseng. J Ginseng Res.

[13] Yang Y., Li J., Zhou S., Ni D., Yang C., Zhang X. (2024). Enhanced immunity effect of Korean red Ginseng capsule: a randomized, double-blind and placebo-controlled clinical trial. J Ginseng Res.

[14] So S.H., Lee J.W., Kim Y.S., Hyun S.H., Han C.K. (2018). Red ginseng monograph. J Ginseng Res.

[15] Li W., Zhuang T., Wang Z., Wang X., Liu L., Luo Y. (2023). Red ginseng extracts ameliorate high-fat diet-induced obesity and insulin resistance by activating the intestinal TGR5-mediated bile acids signaling pathway. Phytomedicine.

[16] Kim S.Y., Seo S.K., Choi Y.M., Jeon Y.E., Lim K.J., Cho S. (2012). Effects of red ginseng supplementation on menopausal symptoms and cardiovascular risk factors in postmenopausal women: a double-blind randomized controlled trial. Menopause.

[17] Wiklund I.K., Mattsson L.A., Lindgren R., Limoni C. (1999). Effects of a standardized ginseng extract on quality of life and physiological parameters in symptomatic postmenopausal women: a double-blind, placebo-controlled trial. Swedish alternative medicine group. Int J Clin Pharmacol Res.

[18] Oh K.J., Chae M.J., Lee H.S., Hong H.D., Park K. (2010). Effects of Korean red ginseng on sexual arousal in menopausal women: placebo-controlled, double-blind crossover clinical study. J Sex Med.

[19] Chung Y.S., Lee I.O., Lee J.Y., Nam E.J., Kim S.W., Kim Y.T. (2021). Effects of Korean red ginseng (Panax ginseng C.A. Meyer) on menopausal symptoms in premenopausal women after gynecologic cancer surgery: a double-blind, randomized controlled trial. J Alternative Compl Med.

[20] Chung T.H., Kim J.H., Seol S.Y., Kim Y.J., Lee Y.J. (2021). The effects of Korean red ginseng on biological aging and antioxidant capacity in postmenopausal women: a double-blind randomized controlled study. Nutrients.

[21] Lee K.J., Ji G.E. (2014). Free-fatty-acid-regulating effects of fermented red ginseng are mediated by hormones and by the autonomic nervous system. J Ginseng Res.

[22] Hyun S.H., Han C.K., So S.H., Park S.K., Park C.K., In G. (2022). Safety of red ginseng and herb extract complex (RHC) in menopausal women: a randomized, double-blind, placebo-controlled trial. J Ginseng Res.

[23] Ministry of Food and Drug Safety (Korea) (2016 Nov 4). Amendment of health functional food code[Internet]. Notification No 2016-72 Cheongju: MFDS.

[24] Cruz E.F., Nina V.J., Figueredo E.D. (2017). Climacteric symptoms and sexual dysfunction: association between the blatt-kupperman index and the female sexual function index. Rev Bras Ginecol Obstet.

[25] Contreras S. (2004). Reliability and validity of the beck depression and anxiety inventories in caucasian Americans and latinos. Hisp J Behav Sci.

[26] Soldatos C.R., Dikeos D.G., Paparrigopoulos T.J. (2000). Athens Insomnia scale: validation of an instrument based on ICD-10 criteria. J Psychosom Res.

[27] Liu S.J., Huang Z.S., Wu Q.G., Liang Y.Y., Miao Y., Miao M.S. (2008). Diagnostic and quantitative criteria of syndrome of fire-heat. Chinese Journal of Modern Drug Application.

[28] The State Administration for Market Regulation of China (2023 Aug 15). Functional food EfficacyTesting and evaluation methods in China.

[29] Shim M.K., Lee Y.J. (2012). Estrogen receptor is activated by Korean red ginseng in vitro but not in vivo. J Ginseng Res.

[30] Zhang L., Chen X., Cheng Y., Chen Q., Tan H., Son D. (2019). Safety and antifatigue effect of Korean red Ginseng: a randomized, double-blind, and placebo-controlled clinical trial. J Ginseng Res.

[31] Chen R. (2023). Interpretation on the 2023 Chinese menopause symptom management and Menopausal hormone therapy Guidelines. Medical Journal of Peking Union Medical College Hospital.

[32] Gilligan D.M., Quyyumi A.A., Cannon R.O. (1994). 3rd. Effects of physiological levels of estrogen on coronary vasomotor function in postmenopausal women. Circulation.

[33] Chen F., Wang L., Zhu Z.Y., Gao C.Y., Wang X.P., Xu Y. (2013). Research progress on the role of endothelin-1 and its receptors in cardiovascular diseases. Chinese General Practice.

[34] Jovanovski E., Peeva V., Sievenpiper J.L., Jenkins A.L., Desouza L., Rahelic D. (2014). Modulation of endothelial function by Korean red ginseng (Panax ginseng C.A. Meyer) and its components in healthy individuals: a randomized controlled trial. Cardiovasc Ther.

[35] Han K., Shin I.C., Choi K.J., Yun Y.P., Hong J.T., Oh K.W. (2005). Korea red ginseng water extract increases nitric oxide concentrations in exhaled breath. Nitric Oxide.

[36] Alblooshi S., Taylor M., Gill N. (2023). Does menopause elevate the risk for developing depression and anxiety? Results from a systematic review. Australas Psychiatry.

[37] Brinton R.D., Yao J., Yin F., Mack W.J., Cadenas E. (2015). Perimenopause as a neurological transition state. Nat Rev Endocrinol.

[38] Klinge C.M. (2017). Estrogens regulate life and death in mitochondria. J Bioenerg Biomembr.

[39] Albert K.M., Newhouse P.A. (2019). Estrogen, stress, and depression: cognitive and biological interactions. Annu Rev Clin Psychol.

[40] Yang L. (2002). A summary of the study on occurrence mechanism of sports fatigue. J Wuhan Inst Phys Educ.

[41] Yang Y., Wang H., Zhang M., Shi M., Yang C., Ni Q. (2022). Safety and antifatigue effect of Korean Red Ginseng capsule: a randomized, double-blind and placebo-controlled clinical trial. J Ginseng Res.

[42] Demir O., Ozalp M., Sal H., Aran T., Osmanağaoğlu M.A. (2020 Mar). The relationship of menopausal symptoms with the type of menopause and lipid levels. Prz Menopauzalny.

[43] Kaya C., Cengiz H., Yeşil A., Ekin M., Yaşar L. (2017 Dec). The relation among steroid hormone levels, lipid profile and menopausal symptom severity. J Psychosom Obstet Gynaecol.

[44] Dijk G.M., Kavousi M., Troup J., Franco O.H. (2015 Jan). Health issues for menopausal women: the top 11 conditions have common solutions. Maturitas.

[45] Ko S.H., Kim H.S. (2020). Menopause-associated lipid metabolic disorders and foods beneficial for postmenopausal women. Nutrients.

[46] Hernández-García D., Granado-Serrano A.B., Martín-Gari M., Naudí A., Serrano J.C. (2019 Oct 28). Efficacy of Panax ginseng supplementation on blood lipid profile. A meta-analysis and systematic review of clinical randomized trials. J Ethnopharmacol.

