# Supplementary material for: Effect of red ginseng beverage on menopausal symptoms in Chinese women: A randomized, double-blind, placebo-controlled clinical trial
Source: J Ginseng Res. 2025 Dec 13;50(2):100931. doi: 10.1016/j.jgr.2025.12.004 (PMC12959274; doi:10.1016/j.jgr.2025.12.004)
Supplement: Multimedia component 1 [file mmc1.docx]

## **Appendix A.** **Method to induce exercise fatigue**

Exercise-induced fatigue was achieved via gradient load exercise, following the standards of a submaximal intensity exercise test. A power bicycle (Ergoline 100) was used as the auxiliary equipment, and the maximum exercise heart rate was calculated as (195 − age) based on each participant’s age.

The specific protocol was conducted as follows: First, a 60-second warm-up period was set on the power bicycle with an initial power of 25 W. After the warm-up, the power of the bicycle was automatically increased in 50 W increments, with each gradient stage maintained for 120 seconds. The exercise was automatically terminated when the participant’s heart rate reached 80% of their maximum heart rate. After termination, the bicycle power was restored to 25 W, and the participants continued to exercise at this intensity for another 60 seconds. Throughout the entire exercise process, the participants’ blood pressure and heart rate were closely monitored to ensure their safety [1,2,3].

**Appendix References：**

1. **Li PF, Feng BX, Shang WY, Zhang WY, Zong PF, Song GQ. Comparative Study of Three Different Incremental Exercises for Evaluating Aerobic Capacity of Cyclist on a Cycle Ergometer. China Sport Science and Technology. 2010;46(2)**:123-125. https://doi.org/10.3969/j.issn.1002-9826.2010.02.021.
2. **Xie SR. The human feeding study on relieving physical fatigue of multi-vitamins amino acid functional beverage[master's thesis].Lanzhou: Lanzhou University; 2016.**

3. Yang Y, Wang H, Zhang M, Shi M, Yang C, Ni Q, et al. Safety and antifatigue effect of Korean Red Ginseng capsule: A randomized, double-blind and placebo-controlled clinical trial. J Ginseng Res. 2022;46(4):543-9. https://doi.org/10.1016/j.jgr.2021.09.001.

****Appendix B. The results of the exercise-induced fatigue test****

As shown in Table S1, compared with baseline, RGB intervention significantly reduced serum creatine kinase (*p* < 0.01), blood lactate levels (*p* < 0.05) and subjective physical strength rating scores (*p* < 0.001). Compared with the placebo group, the RGB group exhibited significant reductions in blood lactate and CK levels (*p* < 0.05), as well as in subjective fatigue scores (*p* < 0.01).

**Table S1** Changes of exercise-induced fatigue indicators before and after the test (‾x±SD)

|  | **RGB group（n=52）** | |  | **Placebo group（n=51）** | |
| --- | --- | --- | --- | --- | --- |
|  | Before test | After test |  | Before test | After test |
| Cortisol (μg/dL) | 10.01±3.38 | 9.43±2.68 |  | 9.76±3.12 | 9.51±3.04 |
| Creatine kinase(U/L) | 92.79±38.95 | 76.90±39.81**# |  | 88.08±39.97 | 94.53±49.08 |
| blood lactate (μmol/mL) | 0.28±0.09 | 0.24±0.09*# |  | 0.28±0.09 | 0.29±0.11 |
| subjective physical scale（score） | 11.12±0.68 | 10.56±0.98***## |  | 11.10±0.78 | 11.12±1.09 |

Comparison within groups**p*<0.05 ***p*<0.01 ****p*<0.001，comparison between groups #*p*<0.05 ##*p*<0.01

****Appendix C.** Report on the intention-to-treat (ITT) analysis**

**Table 1** Baseline characteristics of the two groups before the test ($\bar{x}$±SD)

|  | **RGB group（n=56）** | **Placebo group（n=56）** |
| --- | --- | --- |
| Age (years old) | 53.13±4.76 | 52.38±4.24 |
| BMI(kg/m^2^) | 25.08±3.00 | 24.84±3.12 |
| Systolic pressure（mm/Hg） | 127.61±11.97 | 124.11±13.34 |
| Diastolic pressure（mm/Hg） | 78.05±8.19 | 76.20±9.04 |
| Kupperman index | 28.82±2.59 | 28.55±2.63 |
| Hot flashes and sweating | 8.36±2.19 | 8.29±2.51 |
| Insomnia | 3.04±1.08 | 2.86±1.07 |
| Irritability | 3.46±1.24 | 3.29±1.11 |
| Fatigue | 1.64±0.55 | 1.61±0.49 |
| NO(μmoL/ mL) | 0.07±0.02 | 0.06±0.02 |
| eNOS (ng/mL) | 3.55±1.46 | 3.27±1.31 |
| ET-1(pg/mL) | 20.12±5.21 | 21.30±4.76 |
| FSH(mIU/mL) | 71.36±24.94 | 65.99±23.62 |

**Table 2** Changes of total score of Kupperman index before and after the test (‾x±SD)

|  | **RGB group（n=56）** | |  | **Placebo group（n=56）** | |
| --- | --- | --- | --- | --- | --- |
|  | Before test | After test |  | Before test | After test |
| Total Score of Kupperman index | 28.82±2.59 | 19.96±4.81***### |  | 28.55±2.63 | 28.13±4.46 |
| Hot flashes and sweating | 8.36±2.19 | 5.07±2.70***### |  | 8.29±2.51 | 8.57±2.69 |
| Paresthesia | 2.79±1.36 | 1.86±1.20***# |  | 2.43±1.41 | 2.36±1.33 |
| Insomnia | 3.04±1.08 | 1.54±1.14***### |  | 2.86±1.07 | 2.68±1.10* |
| Irritability | 3.46±1.24 | 1.89±1.23***### |  | 3.29±1.11 | 3.21±1.12 |
| Depressed | 0.88±0.63 | 0.70±0.50**# |  | 0.95±0.52 | 0.95±0.52 |
| Vertigo | 1.13±0.54 | 1.11±0.49 |  | 1.09±0.61 | 1.02±0.56 |
| Fatigue | 1.64±0.55 | 0.89±0.62***### |  | 1.61±0.49 | 1.63±0.59 |
| Bone joint, and muscle pain | 1.02±0.59 | 1.00±0.38 |  | 1.20±0.59 | 1.18±0.64 |
| Headache | 1.21±0.59 | 1.18±0.54 |  | 1.13±0.61 | 1.09±0.58 |
| Cardiopalmus | 1.02±0.40 | 0.93±0.46 |  | 1.04±0.43 | 1.02±0.52 |
| Formication | 0.82±0.61 | 0.66±0.61** |  | 0.80±0.55 | 0.79±0.56 |
| Algopareunia | 1.75±1.15 | 1.64±1.02 |  | 2.04±1.45 | 1.93±1.43 |
| Urinary symptoms | 1.71±1.40 | 1.50±1.16 |  | 1.86±1.26 | 1.71±1.23* |

Comparison within group ***p*<0.01 ****p*<0.001，comparison between groups #*p*<0.05 ###*p*<0.001

**Table 3** Changes of secondary outcomes before and after the test (‾x±SD)

|  | **RGB group（n=52）** | |  | **Placebo group（n=51）** | |
| --- | --- | --- | --- | --- | --- |
|  | Before test | After test |  | Before test | After test |
| **Vasomotor indicators** | | | | | |
| NO(μmoL/ mL) | 0.07±0.02 | 0.08±0.02*## |  | 0.06±0.02 | 0.06±0.02 |
| eNOS (ng/mL) | 3.55±1.46 | 3.33±0.98 |  | 3.27±1.31 | 3.21±1.01 |
| ET-1(pg/mL) | 20.12±5.21 | 18.22±4.95*## |  | 21.30±4.76 | 21.23±4.98 |
| **Psychological assessments** | | | | | |
| Beck depression inventory | 13.89±2.56 | 10.84±2.37***### |  | 13.57±3.01 | 13.70±3.40 |
| Athens insomnia scale | 5.54±1.68 | 4.45±1.33***### |  | 5.79±1.65 | 6.09±1.34 |
| **Lipid profiles** |  |  |  |  |  |
| TC | 6.22±1.22 | 5.36±0.89***# |  | 6.01±1.11 | 5.76±1.14 |
| TG | 1.27±0.66 | 1.37±0.68 |  | 1.28±0.72 | 1.38±0.75 |
| HDL-C | 1.70±0.32 | 1.64±0.36 |  | 1.72±0.31 | 1.65±0.35 |
| LDL-C | 3.53±0.88 | 3.07±0.73***# |  | 3.32±0.83 | 3.40±0.82 |
| **Exercise-induced fatigue indicators** | |  |  |  |  |
| Cortisol (μg/dL) | 9.85±3.36 | 9.43±2.58 |  | 9.82±3.07 | 9.51±2.90 |
| Creatine kinase(U/L) | 93.91±39.04 | 77.53±38.40**# |  | 89.63±40.83 | 93.73±46.87 |
| blood lactate (μmol/mL) | 0.28±0.09 | 0.25±0.09*# |  | 0.28±0.08 | 0.28±0.10 |
| subjective physical scale（score） | 11.13±0.72 | 10.52±0.95***## |  | 11.13±0.81 | 11.11±1.04 |

Comparison within groups **p*<0.05 ***p*<0.01 ****p*<0.001，, comparison between groups #*p*<0.05 ##*p*＜0.01 ###*p*<0.001
